# Polyurethane Composite with Enhanced Mechanical and Damping Properties Filled with Surface-Grafted Hollow Poly(styrene-alt-maleic anhydride) Microsphere

**DOI:** 10.3390/polym18010059

**Published:** 2025-12-25

**Authors:** Rong Xu, Jun Zhang, Jiafeng Tian, Zhiguo Jiang

**Affiliations:** 1 China Academy of Aviation Manufacturing Technology, Beijing 100024, China; xrong@outlook.com (R.X.); tjf18920552307@163.com (J.T.); 2 College of Materials Science and Engineering, Beijing University of Chemical Technology, Beijing 100029, China; jiangzg@mail.buct.edu.cn; 3 High-Tech Research Institute, Beijing University of Chemical Technology, Beijing 100029, China

**Keywords:** polyurethane, styrene-alt-maleic anhydride, microsphere, damping, composite

## Abstract

Nano/microsized organic fillers play an important role in developing new types of polyurethane (PU) composites. In this study, microsized hollow poly(styrene-alt-maleic anhydride) (PSMA) microsphere was grafted with polytetramethylene glycol (PTMG) or 4-butanediol (BDO) and subsequently incorporated into a PU matrix to fabricate composites. For comparison, composites containing pristine hollow PSMA microsphere and neat PU were also prepared. The mechanical properties, damping properties, thermal stability, crystalline structure and water uptake of the composites and neat PU were investigated. The results show that the incorporation of surface-grafted hollow PSMA microspheres could effectively improve the mechanical and damping properties of PU, increasing tensile strength to 18.7 MPa, raising the tanδ peak to 1.05 and broadening the effective damping range (tanδ > 0.3) to 39.1–40.4 °C. Both PU composites and neat PU exhibited three-step decomposition regions. In the first decomposition region, the thermal stability of PU composites was improved slightly except that it was filled with BDO-graft-PSMA microspheres. But in the second and third decomposition regions, all PU composites showed lower thermal stability than neat PU. The introduction of hollow PSMA microspheres also reduced the crystallinity of the PU matrix, which is attributed to the large diameter of the microspheres disrupting crystalline order.

## 1. Introduction

Polyurethane (PU) stands as one of the most versatile polymers and holds the largest global market by production volume [1]. This versatility stems from the exceptional ease with which its physicochemical and mechanical properties can be tailored. They are suitable for a wide range of applications in various industrial sectors, such as automotive and construction, as well as in everyday items like footwear and furniture. Beyond careful selection of raw materials and synthesis methods, composite technology is crucial for engineering PUs with desired or enhanced functionalities and performance, e.g., mechanical properties [2,3], thermal stability [4,5], damping [6], flame retardancy [7] and dielectric property [8]. Fillers commonly used in PU composites include inorganic powders or minerals [4], nano-particles [3], whiskers [9], hollow and solid microspheres [10], carbon nanomaterials [11], polymers [12], cellulose fibers [13], cellulose nanocrystals [14,15] and so on. Inorganic fillers are widely used in PU composites due to their abundance and their ability to enhance specific matrix properties. However, the significant polarity difference between these fillers and the PU matrix results in poor interfacial compatibility, which detrimentally affects the performance of the composites. To address this issue, surface modification of the fillers is typically required prior to incorporation. For example, Dong et al. [16] prepared PU-coated hollow glass microspheres (HGMs) and incorporated them into a waterborne PU matrix. The applied PU layer significantly improved the interfacial compatibility between the HGMs and the matrix. Compared with inorganic fillers, organic fillers generally demonstrate advantages in interfacial compatibility and dispersion stability. Additionally, organic fillers could often be more readily designed or modified to meet specific needs. However, to the best of our knowledge, research utilizing nano- or micro-sized hollow polymer microspheres as organic fillers remains scarce.

Poly(styrene-alt-maleic anhydride) (PSMA) microsphere is a polymer microsphere that could be prepared by the copolymerization of styrene (St) and maleic anhydride (MA) with controlled size and size distribution through traditional suspension [17] or precipitation [18] polymerization. In recent years, novel methods have been developed to prepare PSMA microsphere, such as the photochemical method [19] and stabilizer-free dispersion polymerization [20]. As carboxyl groups could be easily formed by the hydrolysis of anhydride groups, PSMA microsphere could be used as absorbent to uptake metal ions, e.g., Cu^2+^, Ni^2+^, Pb^2+^ and Co^2+^ from aqueous solutions [21,22]. Besides hydrolyzing into carboxyl groups, cyclic anhydride groups could also react with low molecular weight compounds, such as alcohols and amines [19]. Using these reactions, researchers have developed the application of PSMA microspheres as enzyme [23,24] and drug [25,26] carriers. Previous studies have also shown that PSMA microsphere with porous structures has appreciable adsorption capacity for organic vapors and hydrogen, particularly when they were encapsulated with metal ions or conductive polymers [21,27]. Due to the reactive surface that allows easy surface modification or conjugation with targeting moieties, many new applications of PSMA microsphere will be developed.

In the present study, pristine hollow PSMA microspheres, along with their surface-grafted counterparts functionalized with low and high molecular weight diols, were employed as organic fillers to fabricate PU composites. This study aims to examine the enhancement effect of hollow PSMA microspheres on the mechanical and damping properties of PU composites. To the best of our knowledge, this work presents an initial exploration of hollow PSMA microspheres as a functional filler for PU matrices. The results indicate that even at low loadings, these surface-grafted hollow microspheres can contribute to the enhancement of both the mechanical and damping properties of the resulting composites, suggesting their potential as an effective reinforcing filler for PU systems.

## 2. Materials and Methods

### 2.1. Materials

Hollow PSMA microsphere, with a diameter 700–1000 nm and shell thickness 100–150 nm, was kindly supplied by Key Laboratory of Carbon Fiber and Functional Polymers, Ministry of Education, Beijing University of Chemical Technology, Beijing, China. 2-Ethyl-1,3-hexanediol was supplied by KH Neochem Co., Ltd., Tokyo, Japan. Polybutadiene diol (Krasol F 3000) was supplied by TOTAL Cray Valley, Saint-Avold, France. Polytetramethylene glycol (PTMG1000), liquefied methanediphenyl diisocyanate (MDI) (Lupranate MM103C, NCO: 29–30%) were supplied by BASF, Ludwigshafen, Germany. Acetone, 1,4-butanediol (BDO) and p-toluene sulphonic acid were supplied by Fuchen Chemical, Tianjin, China. All the chemicals were used as received.

### 2.2. Surface-Grafting of Hollow PSMA Microsphere

**Figure 1:**
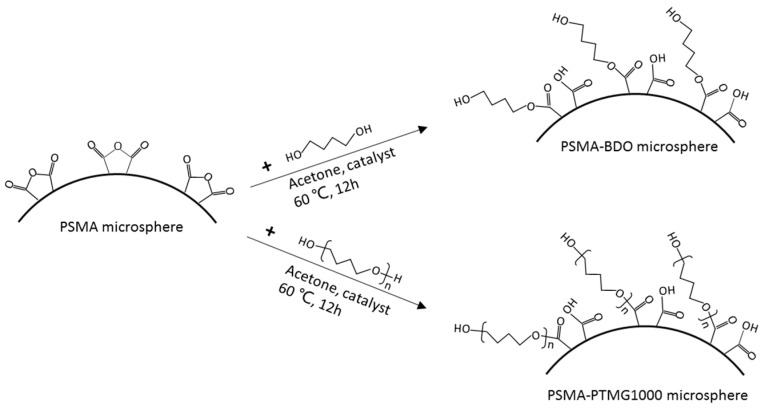
Schematic illustration of surface-grafting of PSMA microsphere with BDO and PTMG1000.

Hollow PSMA microsphere was surface-grafted respectively by BDO and PTMG1000 as shown in Figure 1. The reaction was carried out in a 250 mL flask equipped with a reflux condenser, mechanical stirrer and thermometer (provided by Lichen Instrument Technology Co., Ltd., Shaoxing, China). PSMA hollow microsphere (1.5 g) and BDO (7.0 g) were dispersed in 50 mL of acetone. The mixture was stirred at room temperature for 30 min, and then p-toluene sulphonic acid (0.02 g) was added into the mixture. The reaction mixture was heated under reflux at 60 °C for 12 h, filtered and washed thoroughly with acetone to remove unreacted BDO and dried at 80 °C to remove acetone. The obtained sample was denoted as PSMA-BDO. For PTMG1000 grafting onto PSMA microsphere, the same procedure was repeated with 35.0 PTMG1000 taking the place of BDO, and the sample was denoted as PSMA-PTMG1000.

### 2.3. Preparation of PU Composite Filled with Hollow PSMA Microsphere

PU composites filled with 0.5 wt% of pristine hollow PSMA microsphere were prepared by in situ polymerization. Pristine hollow PSMA microsphere was dispersed in 10.0 g of acetone in a reaction flask with vigorous stirring for 30 min at room temperature. Then the addition of diols (nKrasol F 3000:nPTMG1000:n2-Ethyl-1,3-hexanediol = 0.2:0.8:2.0) was carried out. After being stirred for 30 min, the mixture was heated to 70 °C and stirred for more than 1 h under vacuum condition. This was followed by the addition of liquefied MDI (nNCO:nOH = 1.01:1.0). And then the mixture was stirred for about another 1 h and subsequently cast on a Teflon mold. After eliminating the bubbles under vacuum condition, the resulting reactant was cured at 70 °C for 24 h and denoted as PUP. Same synthesizing steps were repeated to prepare PU composites filled with PSMA-BDO and PSMA-PTMG1000, and the resulting samples were denoted as PUPB and PUPP, respectively. For comparison purposes, a PU containing no microsphere was synthesized in the same manner without the addition of acetone and was denoted as neat PU.

### 2.4. Characterization

Fourier transform infrared (FTIR) spectra were obtained using a Thermo Nicolet Nexus 670 FTIR spectrometer (Thermo Fisher Scientific, Waltham, MA, USA) over the wavenumber range 500–4000 cm^−1^ with a resolution of 4 cm^−1^.

X-ray photoelectron spectroscopy (XPS) was performed on a Thermo SCIENTIFIC ESCALAB 250Xi spectrometer (Thermo Fisher Scientific, Waltham, MA, USA) with a monochromatized Al Kα source (hν = 1486.6 eV). The survey pass energy is 150 eV and energy step size is 1.0 eV.

Thermogravimetric analysis (TGA) was performed under nitrogen atmosphere on a TGA/DSC 1 instrument (Mettler-Toledo, Shanghai, China) from room temperature to 700 °C, at a heating rate of 10 °C·min^−1^.

X-ray diffraction (XRD) was performed on a Rigaku D/max 2500 diffractometer (Rigaku, Tokyo, Japan), using Cu-Kα radiation (40 kV, 50 mA, λ = 0.154 nm) in the 2θ range from 5° to 60° with a step of 0.02° and 2 s/step. The samples were cut into the dimension of 10 mm × 4 mm × 2 mm.

Tensile properties were evaluated on a universal testing machine (XWW-20A, Chengde Jinjian Testing Instrument Co., Ltd., Chengde, China) at a crosshead speed of 200 mm·min^−1^. All tests were conducted at room temperature. Specimens were prepared as dumbbells with dimension of 50 mm × 4 mm × 1 mm in accordance with GB/T 528-2009 [28]. Five replicates were tested for each sample set.

Water absorption test was carried out by immersing the specimens (10 mm × 10 mm × 2 mm) in DI water at room temperature for a total duration of 25 days. Their mass changes were precisely measured and recorded on days 5, 10 and 25. The water absorption rate (*WAR*) was determined using the following equation:

(1) $$WAR=\frac{m_{t}-m_{0}}{m_{0}}\times 100\%$$

where *m*_t_ is the sample weight after immersion in water for a period of time and *m*_0_ is the original weight before immersion.

## 3. Results and Discussion

### 3.1. Characterization of Pristine and Surface-Grafted PSMA

#### 3.1.1. FTIR Spectroscopy Analysis of PSMA

**Figure 2:**
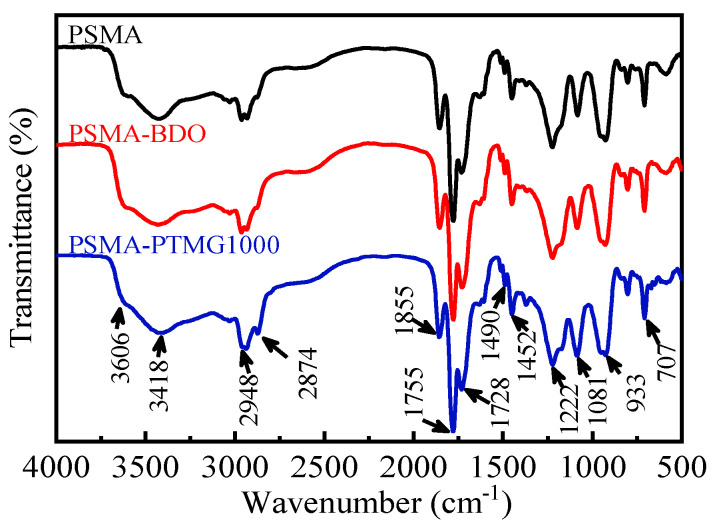
FTIR spectra of PSMA, PSMA-BDO and PSMA-PTMG1000.

The FTIR spectra of PSMA, PSMA-BDO and PSMA-PTMG1000 are presented in Figure 2. The typical absorption bands of PSMA could be found at 1855 cm^−1^ (C=O asymmetric stretching vibration), 1755 cm^−1^ (C=O symmetric stretching vibration), 1222 cm^−1^ (five-membered ring deformation vibration) and 1490 and 1452 cm^−1^ (C=C stretching vibration) [19,20,29]. The absorption bands at 3606 cm^−1^, 3418 cm^−1^ and 1722 cm^−1^ are attributed to free O-H stretching, intermolecular hydrogen bonded O-H stretching and C=O stretching vibration of -COOH, respectively [21,30]. Those typical absorption bands could also be observed from the spectra of PSMA-BDO and PSMA-PTMG1000. The increase in the bands at 3606 cm^−1^, 3418 cm^−1^ and 1722 cm^−1^ observed from the spectra of PSMA-BDO and PSMA-PTMG1000 indicates that BDO and PTMG1000 have been grafted onto PMSA successfully according to the mechanism shown in Figure 1. Moreover, the spectrum of PSMA-PTMG1000 shows higher absorption intensity at 2874 cm^−1^ (symmetric C-H stretching vibration) and 1081 cm^−1^ (C-O-C stretching vibration) than PSMA-BDO because PTMG1000 has more CH_2_ and C-O-C groups than BDO.

#### 3.1.2. XPS Analysis

**Figure 3:**
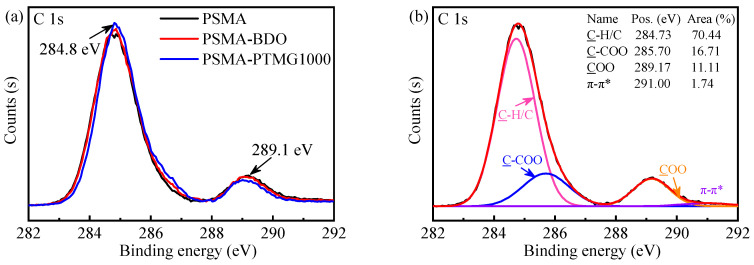

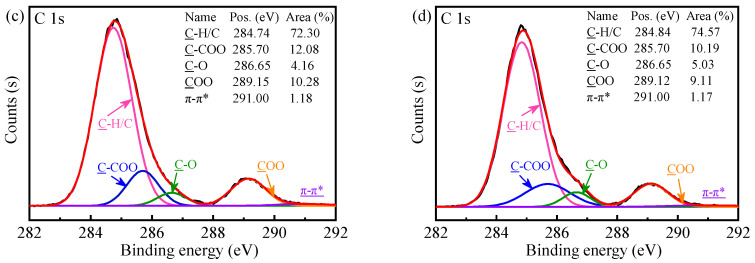
C 1s XPS spectra of the PSMA, PSMA-BDO and PSMA-PTMG1000 (**a**); and peak fittings of PSMA (**b**), PSMA-BDO (**c**) and PSMA-PTMG1000 (**d**).

XPS is a powerful way to identify the atomic composition of solid surfaces and characterize their local chemical environment. Therefore, it was utilized to further determine the chemical species on the surface of the PSMA, PSMA-BDO and PSMA-PTMG1000. The high-resolution C 1s XPS spectra and peak fittings are shown in Figure 3a and Figure 3b–d, respectively. The C 1s spectra (Figure 3a) depict two peaks with binding energies (BE) at around 284.8 and 289.1 eV. The peak at around 284.8 eV corresponds to the hydrocarbons (C-H/C-C/C=C) [31,32], and the shoulder peak at around 289.1 eV is related to the carboxylate carbon (COO), which is obviously from the maleic anhydride groups [31,32]. In comparison with the high-resolution C 1s spectrum of pristine PSMA, those of PSMA-BDO and PSMA-PTMG1000 showed a slight increase at 284.8 eV and a decrease at 289.1 eV. This result indicated that the BDO and PTMG1000 have been successfully grafted on the surface of PSMA, which is consistent with the FTIR result. According to the peak-fitting result of C 1s spectrum of pristine PSMA (Figure 3b), there are another two peaks, assigned to C-COO of MA units at 285.7 eV and to π-π* shake-up satellite from aromatic ring of St units at 291.0 eV [32,33]. From the peak-fitting results of C 1s spectra of PSMA-BDO and PSMA-PTMG1000 (Figure 3c,d), a new peak at 286.65 eV, assigned to C-O from PTMG or BDO [34], could be observed. The C-O component accounts for 5.03% of the total carbon content in PSMA-PTMG1000, which is higher than the 4.16% observed for PSMA-BDO. Furthermore, the ratio of C-O to COO peak areas is 0.55 for PSMA-PTMG1000, which is greater than the ratio of 0.40 for PSMA-BDO. These results quantitatively demonstrate that the flexible polyether segments of PTMG are enriched on the surface of PSMA-PTMG1000, whereas short-chain ester linkages are enriched on the surface of PSMA-BDO.

### 3.2. Characterization of Neat PU and PU Composites

#### 3.2.1. FTIR Spectroscopy Analysis of Neat PU and It Composites

**Figure 4:**
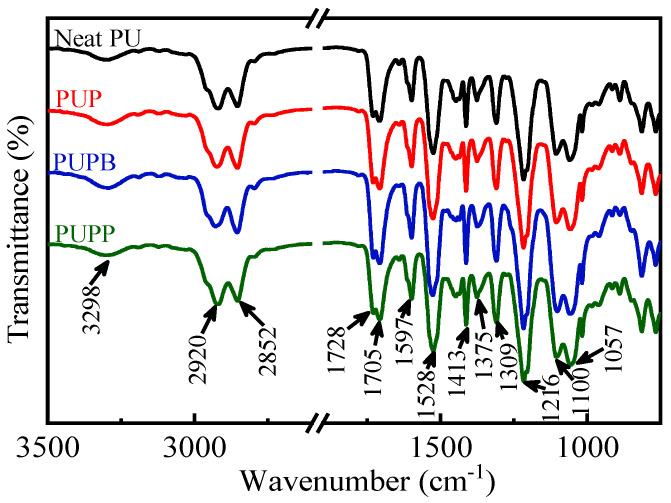
FTIR spectra of neat PU, PUP, PUPB and PUPP.

The FTIR spectra of neat PU and PU composites filled with pristine and surface-grafted PSMA microsphere are presented in Figure 4. The absorption peaks at 3298 cm^−1^ (H-bonded N-H stretching), 1728 cm^−1^ (free C=O stretching), 1705 cm^−1^ (H-bonded C=O stretching) and 1528 cm^−1^ (C-N stretching combined to N-H out-of-plane bending) [35] demonstrate the formation of urethane groups in all the samples. The absorption peaks at 2920 and 2852 cm^−1^ are ascribed to the C-H asymmetric and symmetric stretching of CH_2_ [36]. The absorption peak at 1597 cm^−1^ is ascribed to benzene ring stretching vibration in the MDI segment. Other C-H bond vibrational modes could also be observed at 1413, 1375 and 1309 cm^−1^ [37]. And the presence of C-O-C could be observed at 1216, 1100 and 1057 cm^−1^ [38]. The typical absorption bands of PSMA, like C=O asymmetric stretching vibration at 1855 cm^−1^ and C=O symmetric stretching vibration at 1755 cm^−1^, could not be observed because the amount of pristine and surface-grafted PSMA in PU matrix was only 0.5 wt%.

#### 3.2.2. TGA and DTG Analysis

**Figure 5:**
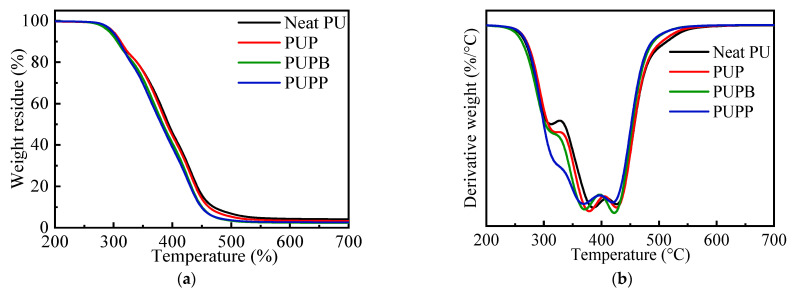
Weight loss (**a**) and derivative (**b**) vs. temperature for neat PU, PUP, PUPB and PUPP.

TGA coupled with DTG was utilized to evaluate thermal behavior [39]. Figure 5 shows the decomposition processes of neat PU and PU composites containing 0.5 wt% of PSMA, PSMA-BDO and PSMA-PTMG1000 microspheres, and Table 1 summarizes the corresponding data.

As shown in Figure 5, neat PU and PU composites have similar thermal decomposition trends and three-step decomposition regions according to the temperature at maximum decomposition rate (T_dmax_), viz. 265–326 °C, 326–400 °C and 400–580 °C. The first step is associated with the decomposition of urethane groups in the hard segment. And the last two steps are associated with the decomposition of polyols in the soft segment and stabilized structures formed in the first step, like carbodiimides, ureas and isocyanurate rings [40]. The pristine and surface-grafted PSMA microsphere greatly affected the thermal decomposition of PU. As shown in Table 1, PUP and PUPP have a relatively higher 5% weight loss temperature (T_5%_) than neat PU, while PUPB has a lower one than neat PU. And all PU composites have a lower 50% weight loss temperature (T_50%_) and T_dmax_ than the neat PU in the second and third decomposition steps. The residue at 700 °C increases in the order of PUPB < PUPP < PUP < neat PU. These results indicate that incorporating either pristine or surface-grafted PSMA microspheres reduces the thermal stability of the PU matrix at elevated temperatures. This decrease is due to the early decomposition of the maleic anhydride (MA) units at approximately 260 °C, which precedes the degradation of the PSMA backbone by about 80 °C [41,42]. This low-temperature step primarily involves the decarboxylation of acid groups and anhydride rings, producing diradicals and releasing carbon monoxide and carbon dioxide [43]. The decarboxylation products are proposed to facilitate free-radical cleavage within the polyol chains, reducing the overall thermal stability of the composites compared to neat PU. This effect is most pronounced above 385 °C, coinciding with the peak carbon dioxide release from PSMA [41]. The presence of acid groups in PSMA, which may further catalyze MA unit decomposition, likely accounts for the relatively lower stability of PUPP and PUPB (with surface-grafted hollow PSMA) versus PUP (with pristine PSMA).

#### 3.2.3. Mechanical Properties

**Figure 6:**
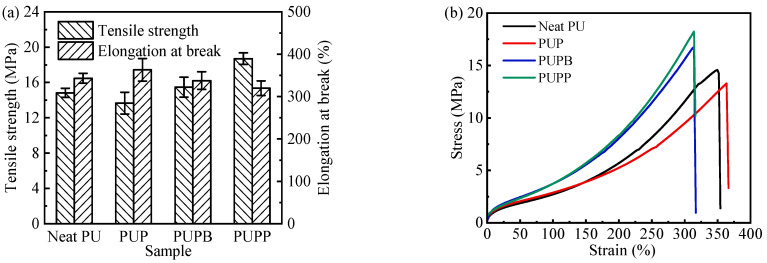
Mechanical properties (**a**) and stress–strain curves (**b**) of neat PU, PUP, PUPB and PUPP.

The mechanical properties and stress–strain curves of neat PU and its composites reinforced with either pristine or surface-grafted hollow PSMA microsphere are shown in Figure 6. An obvious increase in tensile strength (TS) was observed for the composites with surface-grafted microspheres—specifically, 4.3% for PUPB and 26.1% for PUPP—relative to neat PU. This enhancement is ascribed to the cross-linking structure formed at the interface between the grafted microspheres and the PU matrix. By contrast, PUP, which contains pristine (non-grafted) microspheres incapable of such bonding, exhibited a 7.9% decrease in TS. Furthermore, the cross-linking reduces polymer chain mobility, leading to a marginal reduction in elongation at break (EB) for PUPB and PUPP (by 1.7% and 6.7%, respectively). Conversely, the absence of cross-links in PUP allowed for greater chain mobility, resulting in a 5.8% increase in EB.

#### 3.2.4. Water Uptake Property

**Figure 7:**
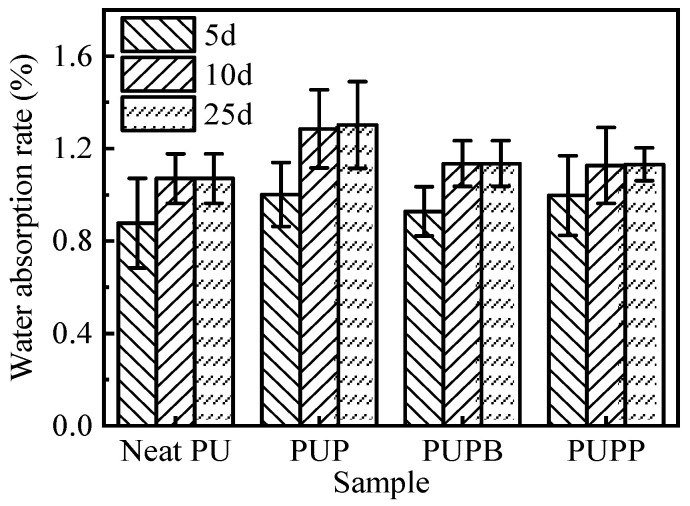
Water absorption rate of neat PU, PUP, PUPB and PUPP.

The water uptake behavior of a material critically influences its applicability in damp environments. Figure 7 compares the water absorption rate (WAR) of neat PU, PUP, PUPB and PUPP after prolonged immersion. All samples exhibited a low WAR (<1.5%) and reached absorption equilibrium after about 10 days. The WAR remained constant between day 10 and day 25. This plateau indicates the achievement of absorption equilibrium. The monotonic approach to saturation without a later abrupt increase is consistent with Fickian-type behavior, suggesting water ingress is primarily diffusion-controlled rather than swelling-controlled [44]. At equilibrium, the WAR increased in the following order: neat PU < PUPP ≈ PUPB < PUP. This trend can be explained by two factors. First, the incorporation of PSMA hollow microspheres (diameter > 700 nm) introduces additional voids that can accommodate water, raising the WAR relative to neat PU. Second, the cross-linking structures in PUPP and PUPB restrict water ingress, resulting in lower absorption compared to the non-cross-linked PUP composite.

#### 3.2.5. Dynamic Mechanical Analysis

**Figure 8:**
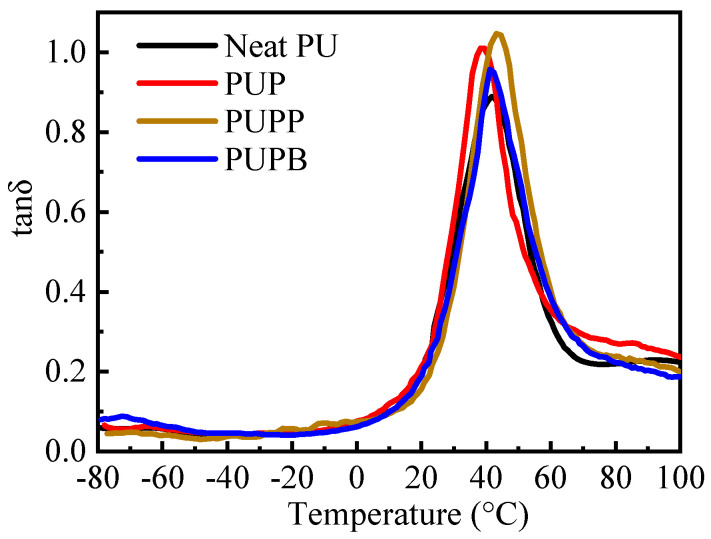
Tanδ vs. temperature for neat PU, PUP, PUPB and PUPP.

PU elastomers generally exhibit superior damping properties due to their characteristic microphase-separated structure of hard and soft segments [45]. Dynamic mechanical analysis was used to investigate the damping properties of neat PU and its composites, with the results shown in Figure 8 and Table 2. Damping, which originates from the dissipation of mechanical energy via molecular chain movements and interfacial friction in composites [6], can be quantified by the loss factor (tanδ) and the temperature range with tanδ > 0.3 [4]. The incorporation of hollow PSMA microspheres enhances damping by increasing interfacial friction (both between the microspheres and the PU matrix and among the microspheres themselves) and providing a large surface area for energy dissipation [46]. Accordingly, as shown in Figure 8 and Table 2, all composites (PUP, PUPB and PUPP) show higher tanδ values (0.95–1.05) and broader effective damping ranges (increased by 17.2%, 3.7% and 6.6%, respectively) than neat PU, confirming the improved damping performance. This broadening of the effective damping temperature range enables promising applications in vibration control, noise mitigation and structural protection across both ambient and elevated temperature conditions. The peak of tanδ generally corresponds to the soft segment glass transition temperature (T_g,SS_) of PU [47]. The cross-linking structure in PUPP restricts chain motion, resulting in a higher T_g,SS_ compared to the other materials. In contrast, the T_g,SS_ of PUP and PUPB decreased by 3.0 °C and 0.8 °C, respectively, relative to neat PU. This reduction indicates that pristine PSMA and PSMA-BDO weaken the interactions among the PU soft segment chains.

#### 3.2.6. XRD Analysis

**Figure 9:**
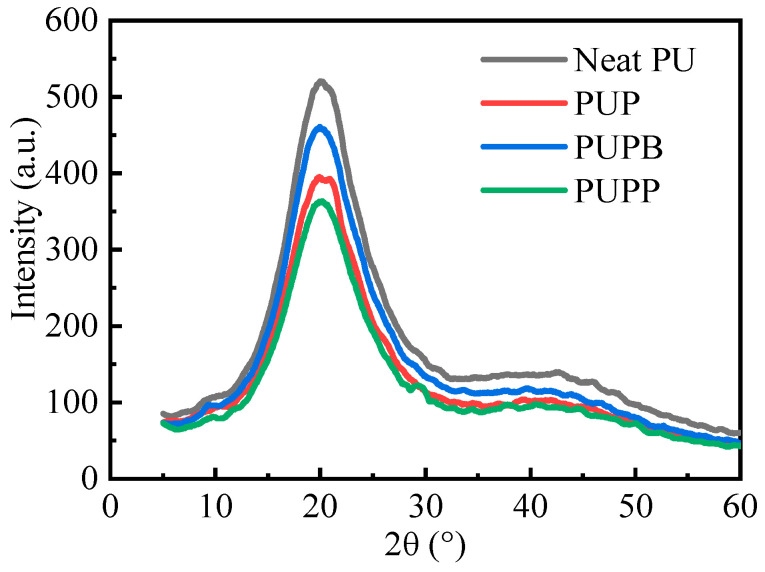
X-ray diffraction spectra of neat PU, PUP, PUPB and PUPP.

X-ray diffraction (XRD) analysis was performed to examine the crystalline structures of neat PU and its composites, as shown in Figure 9. The main diffraction peaks appear at approximately 20°, which corresponds to the MDI-based hard segment crystallinity [48,49]. The sharpness of the peaks decrease in the order of neat PU > PUPB > PUP > PUPP. This decrease demonstrates that the hard segment of neat PU has higher crystallinity and larger crystallite size than PU composites according to Scherrer equation. After the incorporation of pristine and surface-grafted PSMA microsphere into the PU matrix, the distance increased and the interactions decreased between molecular chains. So the molecular chains in hard segment domains are not easy to form crystalline structures. As the microsphere of PSMA-BDO could form cross-linking structure in hard segment domains, the hard segment crystallinity of PUPB is higher than PUP and PUPP. And PUPP has the lowest hard segment crystallinity among the samples. This is because the microsphere of PSMA-PTMG1000 has the longest chain length, which makes the longest distance between the adjacent urethane groups.

## 4. Conclusions

This work advances the field of PU composites by introducing and validating a novel strategy of using surface-grafted hollow PSMA microspheres as a reactive organic filler. The composites containing surface-grafted hollow PSMA microspheres exhibited simultaneous enhancements in both mechanical and damping properties, whereas the composite with pristine hollow PSMA microspheres only showed improved damping. The insights gained here—specifically on the effectiveness of surface grafting for hollow polymer microspheres—provide a valuable framework for the future development of advanced polymer composites where interfacial control is critical.

Although this study was conducted at a low filler loading, it provides initial evidence for the feasibility of this approach. Future work could focus on optimizing filler content, refining the interfacial structure through advanced grafting techniques, leveraging the hollow morphology for additional functionalization and evaluating performance in specific applications. Such efforts would support the progression of these materials from a laboratory-scale concept toward application-oriented composites.

## Figures and Tables

**Table 1:** TGA and DTG data of neat PU and PU composites.

Sample	T_5%_ (°C)	T_50%_ (°C)	Residue at 700 °C (%)	T_dmax-1_ (°C)	T_dmax-2_ (°C)	T_dmax-3_ (°C)
Neat PU	295.6	393.4	4.1	311.4	386.4	427.2
PUP	298.6	390.7	3.3	318.7	378.6	426.6
PUPB	291.6	383.9	2.3	312.3	371.3	422.8
PUPP	297.4	381.1	2.6	320.7	368.9	420.3

**Table 2:** DMA data of PU and PU composites.

Sample	Temperature Range (°C) (tanδ > 0.3)	tanδ_max_	T_g,SS_ (°C)
Neat PU	23.7 to 61.5 (37.8)	0.89	41.9
PUP	24.0 to 68.2 (44.2)	1.01	38.9
PUPB	26.9 to 66.0 (39.1)	0.95	41.1
PUPP	25.3 to 65.7 (40.4)	1.05	43.1

## Data Availability

The original contributions presented in this study are included in the article. Further inquiries can be directed to the corresponding author.
